# Effects of Intermittent Fasting on Liver Steatosis and Fibrosis, Serum FGF-21 and Autophagy Markers in Metabolic Dysfunction-Associated Fatty Liver Disease: A Randomized Controlled Trial

**DOI:** 10.3390/life15050696

**Published:** 2025-04-25

**Authors:** Tugce Ozlu Karahan, Elvan Yilmaz Akyuz, Demet Yilmaz Karadag, Yusuf Yilmaz, Fatih Eren

**Affiliations:** 1 Department of Nutrition and Dietetics, Faculty of Health Sciences, Istanbul Bilgi University, Istanbul 34440, Turkey; tugce.karahan@bilgi.edu.tr; 2 Department of Nutrition and Dietetics, Hamidiye Faculty of Health Sciences, University of Health Sciences, Istanbul 34668, Turkey; elvan.yilmazakyuz@sbu.edu.tr; 3 Institute of Gastroenterology, Marmara University, Istanbul 34854, Turkey; yilmz.demet@gmail.com; 4 Department of Gastroenterology, School of Medicine, Recep Tayyip Erdoğan University, Rize 53100, Turkey; dryusufyilmaz@gmail.com; 5 Department of Medical Biology, School of Medicine, Recep Tayyip Erdoğan University, Rize 53100, Turkey

**Keywords:** intermittent fasting, fibroblast growth factor 21, autophagy, fatty liver, time-restricted eating

## Abstract

Background: This randomized controlled study sought to determine the effect of intermittent fasting on anthropometric measurements, fibroblast growth factor (FGF)-21, and autophagy markers, as well as on hepatic steatosis and fibrosis levels in overweight or obese patients with metabolic dysfunction-associated fatty liver disease (MAFLD). Methods: Patients were randomly assigned into two groups: received a dietary treatment involving 22–25 kcal/kg/day of energy for 8 weeks and followed the same dietary intervention and a 16:8 pattern. The extent of hepatic steatosis and fibrosis was determined using transient elastography on a FibroScan^®^ device. The controlled attenuation parameter (CAP) and liver stiffness measurement (LSM), determined by transient elastography, reflect hepatic steatosis and fibrosis, respectively. In duplicate, serum levels of FGF-21, Beclin-1, and ATG-5 were determined using enzyme-linked immunosorbent assay. Results: The study included 48 patients with a mean age of 48.2 ± 1.4 years (27 female and 21 male). Improvements in anthropometric measurement and CAP and LSM levels and a decrease in serum FGF-21 levels were found in both groups (*p* < 0.05). Changes in the CAP and FGF-21 levels were higher in the energy + time-restricted diet group (*p* < 0.05). Autophagy-related protein (ATG)-5 levels increased only in the energy + time-restricted diet group [(0.74 (0.46–1.29) ng/mL vs. 0.95 (0.73–1.32) ng/mL, *p* = 0.03]. Conclusions: Intermittent fasting was potentially practical in the management of MAFLD. In particular, changes in FGF-21 and ATG-5 levels indicate the potential of intermittent fasting to regulate metabolic processes and autophagy. However, methodological limitations should be taken into consideration when interpreting the study results.

## 1. Introduction

Metabolic dysfunction-associated fatty liver disease (MAFLD), formerly known as non-alcoholic fatty liver disease (NAFLD), is characterized by fat deposition in the liver, is associated with obesity, diabetes, hypertension, and dyslipidemia, and continues to increase in prevalence [1]. MAFLD is seen in approximately one-quarter of the adult population and has a prevalence of 45.5% in Turkey; it poses a substantial economic burden on the healthcare systems across the world [2]. Low awareness, gaps and failures in treatment, and increasing prevalence make MAFLD a significant public health problem [3].

Despite the increasing burden of the disease, there was no treatment approved by the United States Food and Drug Administration (FDA) until the recent approval of resmetirom. The approval of resmetirom, an oral, liver-targeted, thyroid hormone receptor beta-selective agonist, represents a promising therapeutic option for adults with moderate to advanced liver fibrosis [4]. Nevertheless, lifestyle modifications remain the gold standard in treatment. Different dietary components influence the progression and development of MAFLD, and nutrition’s role in treating MAFLD has been extensively studied [5]. Published guidelines for managing the disease have emphasized losing at least 3–5% of body weight to improve hepatic steatosis and further weight loss (7–10%) to enhance histological parameters [6,7]. Considering the challenges of patient compliance with energy restrictions, intermittent fasting diets have recently gained popularity as an alternative approach. Time-restricted eating (16/8) is one of the most used and tested fasting protocols [8]. The 16/8 time-restricted diet (TRF) model is a dietary pattern in which daily energy intake is restricted to 8 h, with no calorie intake for the remaining 16 h. This pattern can positively affect metabolic health by supporting circadian rhythm [9]. Metabolic benefits of TRF include improved insulin sensitivity, reduced inflammation, and control of body weight [10]. In particular, the 16/8 model was found to be more sustainable compared to other intermittent fasting protocols and was evaluated as a potential strategy for managing diseases such as obesity, metabolic syndrome, and MAFLD [11,12]. Previous studies have found significant improvements in the body mass index (BMI) and serum lipid profiles with a time-restricted diet. Thus, the intermittent fasting protocol has been hypothesized to be a potentially effective intervention in MAFLD [13,14,15].

One of the mechanisms by which intermittent fasting counteracts disease processes is the activation of cellular stress response signaling pathways that stimulate autophagy [16]. In recent years, increasing evidence suggests that autophagy is regulated during eating–starving cycles under physiological conditions [17,18]. Disruption of autophagic flux due to lipid accumulation in hepatocytes is one of the factors leading to MAFLD progression. High-fat diets in mice induce MAFLD, block hepatic autophagy, and lead to oxidative stress and mitochondrial dysfunction [19]. These findings support the molecular basis of autophagy induction in the prevention or regression of MAFLD [20]. A recent study in mice showed that overnight fasting increases the expression of autophagy-related genes, including autophagy-related protein (ATG)-7, and regulates hepatic autophagy-mediated lipid metabolism [21]. Another study in mice showed that the impaired autophagic flux induced by a high-fat diet was reversed by intermittent fasting [22].

The mechanism between intermittent fasting and hepatic autophagy activates fibroblast growth factor (FGF)-21 signaling. Mechanistically, FGF-21-activated protein kinase A (PKA) mediates nuclear localization and phosphorylation of Jumonji domain containing 3 (JMJD3) to activate autophagy transcriptionally. Under nutrient deprivation, the fasting-induced hepatokine JMJD3 is activated by FGF-21 and epigenetically up-regulates global autophagy network genes [21]. In clinical studies, elevated serum levels of FGF-21 have been associated with obesity, metabolic syndrome, and diabetes [23], which are components of MAFLD. FGF-21 has been identified as a key regulator of glucose, lipids, and overall energy balance [24]. Endogenous FGF-21 has been shown to facilitate insulin-independent glucose uptake in adipocytes and promote lipolysis, which reduces fat storage [25,26]. Exogenous FGF-21 administration has been shown to reduce glucose and triglyceride plasma levels and encourage weight loss in experimental models [26]. Further, serum FGF-21 levels were associated with MAFLD and increased significantly and gradually in parallel with hepatic steatosis scores [27].

Based on the available scientific evidence, the role of the TRF (16:8) model in managing MAFLD through autophagy is still not fully understood. Therefore, comprehensive studies are needed to examine the effects of TRF on autophagy activation, FGF-21 signaling pathway, and lipid metabolism. Our study aims to evaluate the potential therapeutic effects of TRF on MAFLD and its relationship with the autophagy mechanism and how nutrition-based interventions can be used more effectively in disease management. The findings may provide significant contributions to the scientific literature on treatment strategies for MAFLD and shed light on the development of new nutritional approaches in clinical practice.

Based on the available scientific evidence, the role of the TRF (16/8) model in managing MAFLD through autophagy is still not fully understood. The present study sought to investigate the effect of the intermittent fasting protocol (16/8) on hepatic steatosis and fibrosis parameters in patients with MAFLD. We also analyzed serum FGF-21 and autophagy parameters to explain the mechanism behind the effect of intermittent fasting on the course of the disease. Our study aims to evaluate the potential therapeutic effects of TRF on MAFLD and its relationship with the autophagy mechanism and how nutrition-based interventions can be used more effectively in disease management. This study will determine the potential therapeutic effects of TRF on MAFLD and its relationship with the autophagy mechanism and evaluate how nutritional interventions can be used more effectively in disease management.

## 2. Materials and Methods

### 2.1. Patients

This study was approved by the Ethics Committee of Marmara University School of Medicine (No: 09.2022.707) and was registered at ClinicalTrials.gov (NCT06664684). Informed consent was obtained from each patient included in the study. This is a prospective non-drug intervention study conducted by the same gastroenterologist on patients diagnosed with MAFLD. The sample size was calculated using G*Power 3.1.9.7 software based on the expected difference in liver stiffness measurement (LSM) scores (approximately 1.03 kPa) and an estimated standard deviation of ~1.3 kPa [28]. For the comparison between two independent groups, the effect size was determined to be 0.8. With a significance level of 5% and a statistical power of 80%, a minimum of 21 participants per group, and a total of 42 participants, was considered sufficient for the study. In our study, recruitments were carried out between May 2022 and January 2023. The study included patients who were diagnosed with MAFLD, were aged 18–65 years, had a BMI ≥ 25 kg/m^2^, and had a stable body weight (<5 kg weight loss or gain) over the last 3 months preceding the start of the study, and signed the informed consent form. Exclusion criteria were patients with an average daily alcohol consumption of >20 g for females and >30 g for males; pregnant or lactating women; patients with ischemic heart disease or heart failure, chronic inflammatory diseases, chronic viral infections, cancer, moderate-to-severe kidney disease, uncontrolled hypertension, and eating disorders; those with a history of bariatric surgery; and those on insulin due to increased risk of hypoglycemia. The patients were diagnosed with fatty liver by a gastroenterologist specialized (Y.Y.) in liver diseases based on Fibroscan results. Individuals with fatty liver disease diagnosed by vibration-controlled transient elastography are diagnosed with MAFLD if at least one of the following three conditions is present: overweight/obesity, type 2 diabetes mellitus (T2DM), or underweight or normal weight with at least two metabolic risk abnormalities [waist circumference, blood pressure, triglycerides, high-density lipoprotein cholesterol (HDL-C), prediabetes, insulin resistance, high-sensitivity C reactive protein (CRP)] [29]. All of our patients were diagnosed with MAFLD because their BMI was above 25 kg/m^2^ in addition to the detection of fatty liver.

**Figure 1:**
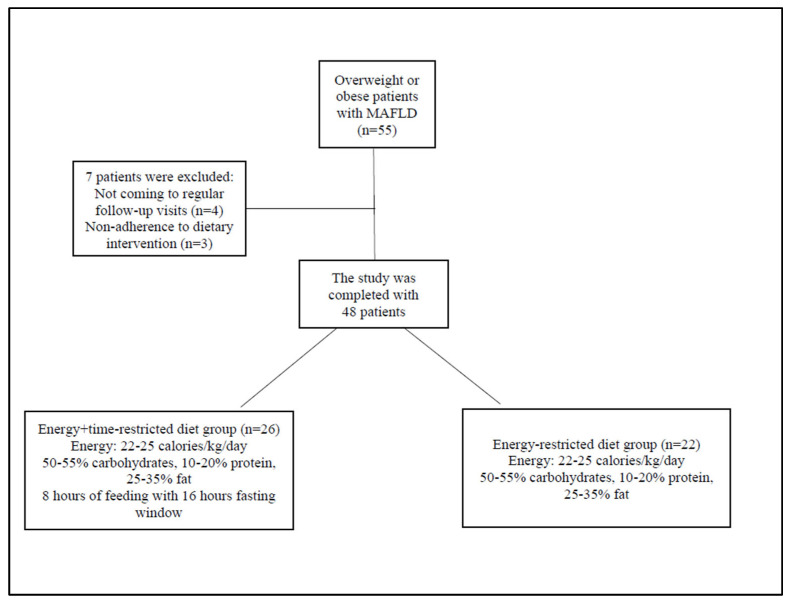
Design of the study.

Exclusion criteria were based on questionnaire-based patient declaration for other diseases outside the field of gastroenterology. Considering possible losses, the study started with 55 patients and later dropped seven patients who failed to attend regular follow-up visits during the intervention period or failed to comply with dietary treatments. Thus, the study was completed with 48 patients (Figure 1).

### 2.2. Study Design

**Figure 2:**
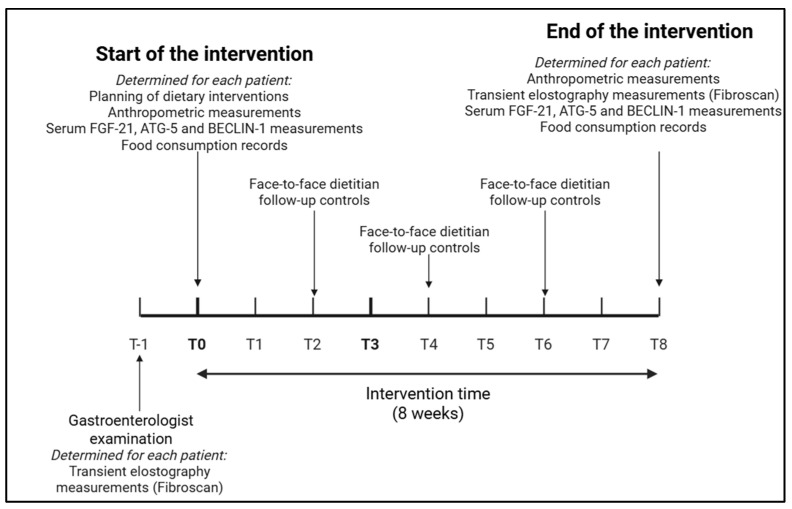
Flowchart of the study.

Patients who voluntarily agreed to participate in the study were randomly assigned into two groups. Patients enrolled in the study were referred for an initial consultation with a dietitian following their diagnosis by a gastroenterologist. During this consultation, they were informed about the study, and their voluntary consent was obtained. Upon obtaining consent, the patients were randomly assigned to two groups. The sealed envelope method was used to ensure that the randomization process was carried out in a standardized manner. Envelopes labeled Group 1 and Group 2 were prepared according to the number of patients participating in the study. The envelopes were mixed in a random order, and when each patient was recruited, one of the sealed envelopes was randomly selected and opened. The energy-restricted diet group followed an energy-restricted diet for 8 weeks, while the energy + time-restricted diet group followed the same diet and a 16:8 eating pattern and restricted food intake to an 8 h window each day. Both groups were given the same dietary content to better statistically elucidate the eating window’s positive, negative, or neutral effect (Figure 1). The effectiveness of the intervention was assessed using anthropometric and biochemical measurements, and Fibroscan^®^ investigations were performed before the intervention (T0) and after the intervention (T8) The flow diagram of the study is given in Figure 2. Fibroscan (Y.Y.) and biochemical measurements (F.E. and D.Y.K.) were performed by investigators who did not know which intervention group the patients were in. Only control visits and dietary counseling were provided by the principal investigator (T.O.K.). Still, as mentioned above, the principal investigator did not perform the measurements to determine the effectiveness of the interventions. Except for serum FGF-21 and autophagy parameters, fasting plasma glucose, alanine aminotransferase (ALT), aspartate aminotransferase (AST), gamma-glutamyl transferase (GGT), total cholesterol, triglyceride, low-density lipoprotein cholesterol (LDL-C) and HDL-C levels, which are routinely measured in patients, were obtained from patient files as the values in the last 1 month before and after the intervention.

### 2.3. Dietary Interventions

The energy-restricted diet group followed an 8-week-long dietary treatment involving 22–25 kcal/kg/day based on ideal body weight [30]. The diets were planned based on current guidelines, manuals, systematic reviews, and meta-analyses published in recent years on MAFLD [6,7,31]. In this diet, carbohydrates constituted 50–55% of total energy intake, proteins 10–20%, and fats 25–35%. The content of the diets was tailored to each patient, considering various factors such as sex, age, and physical activity status.

Patients in the energy + time-restricted diet group followed the same dietary intervention and a 16:8 eating pattern. They were instructed to restrict their energy intake to an 8 h window and not to consume energy-containing foods or drinks during the remaining 16 h. Patients were allowed to consume energy-free beverages such as water, coffee, and tea during fasting. The timing of the eating window during the day varied according to patients’ lifestyles and habits. However, considering the importance of nocturnal fasting, the eating window in all patients started at 10:00–12:00 a.m. and ended at 6:00–8:00 p.m. The energy-restricted diet group did not follow any time restrictions in planning main meals and snacks.

Patients did not receive any advice on physical activity during the intervention. None of the patients exercised regularly at baseline, which did not change during the intervention.

### 2.4. Monitoring of Dietary Interventions

During the 8-week intervention period, the researcher conducted in-person follow-up checks with patients every 2 weeks. During the in-person visits, patients underwent anthropometric measurements, had their diet reviewed, and their food intake recorded for 3 days, including 1 day of the weekend, to determine daily energy intake and dietary components. The Nutrition Information System (BeBIS) 8.1 software calculates the average daily energy intake and nutritional components. In-person checks were complemented with daily phone calls to monitor and ensure patients’ compliance with their diets closely.

### 2.5. Anthropometric Measurements

Patients’ heights were measured using a stadiometer (Seca 769), barefoot, feet side by side, and head in the Frankfort plane. Body composition was determined using the bioelectrical impedance method (Tanita MC 780 P). Further, waist and hip circumference was measured using a nonstretchable tape measure. These measurements based on bioelectrical impedance analysis allow for determining body weight, BMI, body fat weight and percentage, and fat-free mass. Analyses were performed by paying due attention to prerequisites for accuracy, including fasting for at least 4 h before analysis, avoiding measurements during menstruation, removing all metal jewelry, and performing the analysis at normal room temperature.

### 2.6. Transient Elastography

The extent of hepatic steatosis and fibrosis was determined using transient elastography on a Fibroscan 502 Touch^®^ device(Echosens SA, Paris, France). All Fibroscan measurements were performed following the manufacturer’s instructions as specified previously [32]. A reliable measurement was defined as having at least ten valid measurements with an interquartile range–median ratio ≤ 0.3 [33]. Hepatic steatosis and fibrosis were defined using controlled attenuation parameters (CAPs) and LSM. LSM measurement ranged between 2.5 and 75 kPa, and the CAP measurement, which indicates steatosis, ranged between 100 and 400 dB/m [34].

### 2.7. Determination of Serum FGF-21, ATG-5, and Beclin-1

Blood samples were collected from patients after 12 h of fasting. The samples were centrifuged for 15 min and stored at −80 °C after serum separation until analysis. Serum FGF-21, Beclin-1, and ATG-5 were analyzed using enzyme-linked immunosorbent assay (ELISA) kits, following the manufacturer’s protocols (Human FGF-21 ELISA, Biovendor, Brno, Czech Republic; Human BECN1 [Beclin-1] ELISA Kit, ElabScience, Houston, TX, USA; Human Autophagy protein 5 [ATG5] ELISA Kit, MyBioSource, Inc., San Diego, USA) and in duplicate. All samples were processed with the investigator blind to the clinical status of the patients. Intra- and interassay coefficients of variability were (<2% and <3%), (<7% and 9%), and (<6% and <5%) for FGF-21, ATG, and Beclin-1, respectively. The detection limit of ATG-5 human serum ELISA KİT was 50 ng/mL–0.78 ng/mL. The minimum detectable Human ATG-5 (sensitivity) was up to 0.1 ng/mL. The limit of detection (LOD) was calculated from the real FGF-21 values in wells and is 7 pg/mL. The sensitivity of the Beclin ELISA Kit was 0.10 ng/mL; the detection range of the Beclin ELISA Kit was 0.16–10 ng/mL.

### 2.8. Statistical Analysis

Study data were analyzed using SPSS 28.0 software suit. Statistical significance was set at *p* < 0.05 for all analyses. Data were checked for normality of distribution using the Kolmogorov–Smirnov test. Categorical variables were analyzed using the chi-square test. Median (Quartile 1–Quartile 3) values were used in descriptive statistics. The Mann–Whitney U test was used to determine whether the baseline values were similar between the two groups before the intervention. The before/after comparison of the quantitative data was performed using the Wilcoxon test. The differences in the changes in quantitative data (the value obtained by subtracting the values at time T0 from the value at time T8) between the two groups after the intervention were compared using the Mann–Whitney U test. The correlation between the variables was assessed using Spearman’s correlation.

## 3. Results

### 3.1. General Characteristics of the Participants

The study included 48 patients with MAFLD (27 female and 21 male) with a mean age of 48.2 ± 1.4 years. Of the patients, 22 (45.8%) followed the energy-restricted diet only, while 26 (54.2%) followed the energy-restricted + time-restricted diet. At T0, there was no difference between the two groups in terms of age, gender, energy and nutrient intake, anthropometric, biochemical and Fibroscan measurements (*p* > 0.05). The fact that the general characteristics of the patients participating in the study were similar in both groups contributed positively to the analyses performed.

### 3.2. Effect of Interventions on Anthropometric and Routine Biochemical Measurements

Changes in anthropometric, biochemical, and Fibroscan measurements of the patients are given in Table 1. Patients in both the energy + time-restricted diet group and the energy-restricted diet group achieved a significant decrease in their body weight, BMI, waist circumference, body fat mass, body fat percentage, and fat-free mass compared with those of T0 (*p* < 0.05). However, changes in anthropometric measurements (body weight, BMI, waist circumference, hip circumference, body fat mass, body fat percentage, body weight loss, and body fat weight loss percentage) were higher in the energy + time-restricted diet group (*p* < 0.05). On the other hand, the change in waist–hip ratio and fat-free mass of the patients did not differ significantly between the groups (*p* > 0.05).

In addition, LDL-C, total cholesterol, ALT, and GGT levels decreased significantly in both the energy-restricted and the energy + time-restricted diet groups compared to the pre-intervention period (*p* < 0.05). The decrease in AST levels was significant only in the energy + time-restricted diet group (*p* < 0.001). However, HDL-C levels did not change significantly in both groups compared to the pre-intervention period (*p* > 0.05).

### 3.3. Effect of Interventions on Fibroscan Measurements

**Figure 3:**
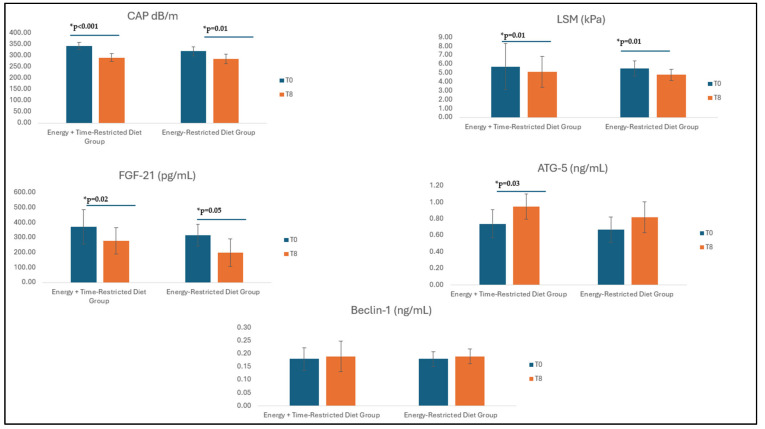
Analysis of biochemical and Fibroscan measurements after intervention. Data are expressed as medians (95% confidence interval for median). Wilcoxon analysis was performed. Statistical significance, * *p* < 0.05, significant *p* values are indicated in bold. CAP: controlled attenuation parameter; LSM: liver stiffness measurement; ATG-5: autophagy-related protein-5; FGF-21: fibroblast growth factor-21. The blue color indicates T0 time, and the orange color indicates T8 time.

At T8, patients in both the energy-restricted diet group [319.5 (296.7–371.5) dB/m vs. 285.0 (268.2–353.7) dB/m, *p* = 0.01] and the energy + time-restricted diet group [343.5 (297.0–363.2) dB/m vs. 291.5 (254.5–324.2) dB/m, *p* < 0.001] achieved a significant decrease in CAP levels which indicates hepatic steatosis (*p* < 0.05). The change in CAP values at T8 was significantly higher in the energy + time-restricted diet group (*p* = 0.04). A decrease in LSM levels which indicates hepatic fibrosis was observed in both groups compared to pre-intervention [5.5 (4.2–6.2) kPa vs. 4.8 (3.9–6.5) kPa, *p* = 0.01 for the energy-restricted diet group; 5.7 (4.6–6.9) kPa vs. 5.1 (4.3–6.3) kPa, *p* = 0.01 for the energy + time-restricted diet group] (Figure 3). However, the change in LSM values did not differ significantly between the two groups (*p* > 0.05).

### 3.4. Effect of Interventions on Serum FGF-21 and Autophagy Markers

As for the biochemical findings analyzed, FGF-21 levels decreased in both energy-restricted [316.9 (135.0–431.7) pg/mL vs. 199.1 (109.8–459.6) pg/mL, *p* = 0.02] and energy + time-restricted diet groups [371.4 (277.7–505.1) pg/mL vs. 278.1 (94.8–331.6) pg/mL, *p* = 0.05]. The change in FGF-21 was higher in the energy + time-restricted diet group (*p* = 0.03). ATG-5 levels were significantly increased at T8 only in the energy + time-restricted diet group [0.74 (0.46–1.29) ng/mL vs. 0.95 (0.73–1.32) ng/mL, *p* = 0.03], while Beclin-1 levels did not change significantly in both groups (*p* > 0.05) (Figure 2).

### 3.5. Correlation Between FGF-21, Autophagy Markers, and Liver Disease Parameters

The relationships between FGF-21 and the levels of ATG-5 and Beclin-1 were examined prior to the intervention. A significant positive correlation was found between serum FGF-21 and ATG-5 (R = 0.343, *p* = 0.02), as well as between BECLIN-1 and ATG-5 (R = 0.342, *p* = 0.02) (Table 2).

Post-intervention correlations among FGF-21, autophagy markers, and liver disease parameters were examined. The percentage of body weight loss was found to be positively correlated with changes in CAP (R = 0.355, *p* = 0.01) and LSM (R = 0.364, *p* = 0.01). However, no significant associations were observed between changes in FGF-21 or autophagy markers and changes in CAP or LSM (*p* > 0.05) (Table 3).

### 3.6. Dietary Intake of Patients and Changes in Energy and Dietary Intake with Interventions

We also compared calorific and dietary component intake among patients at T0 and T8. Results at T8 showed that patients in both the energy + time-restricted diet group and energy-restricted diet group achieved a decrease in their daily energy intake, an increase in the proportion of energy from carbohydrates and protein, and a reduction in the proportion of energy from fat (*p* < 0.05). Further, the intake of fat and saturated fat decreased, while the intake of fiber and fructose increased among all patients (*p* < 0.05). Changes in energy and dietary components did not differ between the groups (*p* > 0.05) (Table 4).

## 4. Discussion

Although weight loss has a well-documented role in managing MAFLD, the content and timing of dietary interventions remain controversial. Considering the difficulties in patient compliance with nutritional treatments, intermittent fasting diets have recently been proposed as an alternative approach [8]. Moreover, assuming the role of autophagic dysfunction in the pathogenesis of MAFLD, intermittent fasting emerges as an even more interesting technique, as it is considered to stimulate autophagy [10]. Thus, the present study sought to assess the effect of the intermittent fasting protocol on hepatic steatosis and fibrosis parameters in patients with MAFLD. Furthermore, the mechanism behind the impact of dietary interventions was examined by analyzing serum FGF-21 and autophagy parameters.

The results of this study showed that changes in anthropometric measurements—body weight, BMI, waist circumference, hip circumference, body fat mass, body fat percentage, body weight loss, and body fat weight loss—were significantly higher in the energy + time-restricted diet group than in the energy-restricted diet group. The importance of reduction in visceral obesity, which is directly related to the severity of MAFLD, should be emphasized in weight loss and improvements in anthropometric parameters [35]. Consistent with these findings, Koda et al. [36] reported visceral fat accumulation as the most important marker of hepatic steatosis severity. Weight loss, focusing on reducing visceral fat, was accompanied by improvements in parameters associated with the severity of hepatic steatosis, inflammation, and fibrosis in patients with MAFLD [36]. Our study found that the change in CAP which indicates hepatic steatosis was significantly higher in the energy + time-restricted diet group than in the energy-restricted diet group. A study similar to ours investigated the efficacy of fasting for 16 h/day without energy restriction for 12 weeks in improving visceral adiposity and steatosis compared with standard care and found a higher decrease in CAP values in the intermittent fasting group (n = 10) than in the standard care group (n = 13) and no significant difference in LSM values within and between the groups compared with baseline [37]. Similarly, the median value of LSM change which indicates hepatic fibrosis in our study tended to be higher in the energy + time-restricted diet group, but this difference was insignificant. Baseline median LSM values based on Fibroscan cutoff points indicate that most patients had minimal hepatic fibrosis. This may explain the absence of a significant difference between the groups regarding the rate of decline in fibrosis.

FGF-21 has recently drawn increasing interest as a therapeutic target for obesity-related metabolic disorders, including MAFLD, mainly because of its effect on lipid and carbohydrate metabolism [38,39]. Most observational studies have reported elevated hepatic or circulating levels of FGF-21 in patients with MAFLD [39,40]. Most imaging-based studies have shown a positive correlation between FGF-21 concentrations and liver fat content [39,41]. In contrast, data on specific histologic lesions are scarce and conflicting. Some authors have shown higher FGF-21 concentrations in more severe hepatic steatosis [27], inflammation, or fibrosis. In contrast, other authors have reported similar FGF-21 levels in groups with different severity levels of steatosis [42], inflammation, and fibrosis [43,44]. FGF-21 levels have been shown to increase in MAFLD patients, which is thought to be related to the metabolic stress response in hepatocytes [27,40]. Moreover, data from studies investigating the effect of lifestyle interventions on FGF-21 also remains limited and conflicting [39]. One study (n = 153) found FGF-21 levels to be higher in patients with excess body weight than in normal-weight individuals. Energy restriction treatment for weight loss was found to cause a significant decrease in circulating FGF-21 levels compared with baseline values. Low-energy and very low-energy ketogenic diet regimens had no difference in FGF-21 levels [45]. Dushay et al. (2010) [40] showed that patients with MAFLD had higher levels of circulating and hepatic FGF-21 than lean control patients and that short-term interventions such as ketogenic diets had no effect on FGF-21 [40]. Further, a randomized controlled study on overweight and obese individuals with MAFLD reported that an energy-restricted diet affected neither circulating FGF-21 levels nor MAFLD [46]. Our study, however, found a significant decrease in FGF-21 levels compared with T0 values in both the energy + time-restricted diet group and the energy-restricted diet group.

Correlation analyses conducted in our study revealed a positive relationship between pre-intervention levels of FGF-21 and ATG-5. These findings support the notion that FGF-21 may act as a stress biomarker in MAFLD and could exhibit resistance-related effects similar to those observed in insulin resistance associated with diabetes [47]. The concurrent increase in ATG-5 levels with rising FGF-21 concentrations may be explained by the activation of autophagy in hepatic cells as an adaptive response to metabolic stress. This suggests that ATG-5 plays a role in mitigating intracellular damage. Nevertheless, current literature on this topic remains limited and warrants further investigation. In the present study, the change in FGF-21 levels was significantly higher in the energy + time-restricted diet group than in the energy-restricted diet group. Recent data suggest intermittent fasting may affect obesity-induced FGF-21 resistance [48,49]. Because the FGF-21 molecule has a circadian rhythm disrupted by a high-fat diet, intermittent fasting has been claimed to have the benefit of rebalancing FGF-21 release and, thus, potentially preventing obesity [49]. Zhu et al. (2016) [50] demonstrated that FGF-21 can improve multiple metabolic parameters involved in the pathogenesis of MAFLD by promoting autophagy [50]. FGF-21 can accelerate the regeneration of damaged liver by promoting autophagy in liver cells through the AMPK/mTOR signaling pathway. Mechanistically, this is closely associated with increased FGF-21 expression, enhanced AMPKα phosphorylation, and stimulation of autophagy. FGF-21 can also induce autophagy by promoting SIRT1 expression [51]. Epigenetic studies revealed that the FGF-21-JMJD3 signaling pathway links nutrient deficiency to hepatic autophagy. The autophagy defect in FGF-21-KO mice strongly suggests that physiological levels of hepatic FGF-21 during fasting may activate autophagy in an autocrine/paracrine manner [21]. These findings indicate that the FGF-21 molecule may be beneficial in treating MAFLD through its autophagic action [52]. Consistent with these findings, our study found a significant increase in ATG-5 levels, autophagy marker, in the energy + time-restricted diet group compared with T0 values. However, Beclin-1 levels did not change significantly in both groups. Correlation analyses did not reveal any significant association between changes in FGF-21 and ATG-5 levels and changes in LSM and CAP. However, a significant correlation was identified between the percentage of body weight loss and changes in both LSM and CAP. These results underscore the pivotal role of weight reduction in the treatment of MAFLD and highlight the need for further studies to elucidate the mechanistic effects of intermittent fasting on the disease.

In recent years, a series of studies have focused on the role of ATG-5 in the development of liver fibrosis. This molecule plays a critical role in the autophagy process as it is involved in the formation of autophagosomes. Reports suggesting that ATG-5 could be used as a therapeutic target in the treatment of liver fibrosis are mostly based on studies conducted in mice or cell cultures [53,54]. On the other hand, Beclin-1 plays a key role in the coordination of autophagy and apoptosis processes. A recent study involving 74 individuals determined that serum Beclin-1 levels were associated with severe liver fibrosis [53]. In contrast, another study conducted on 80 individuals, including a healthy control group, found that serum ATG-5 levels were significantly lower in individuals diagnosed with hepatocellular carcinoma compared to the control group. As a result of this study, it was suggested that serum Beclin-1 and ATG-5 could serve as novel biomarkers for predicting the risk of hepatocellular carcinoma [55]. Since autophagy operates in the cytoplasm, its influence on secretory proteins—those destined for the plasma membrane or extracellular space and typically synthesized and directed within the endoplasmic reticulum—has been relatively overlooked in the past. However, increasing evidence has revealed that autophagy significantly regulates protein secretion through various mechanisms. Firstly, autophagy is closely involved in the unconventional secretion of leaderless proteins—a pool of proteins targeted for extracellular release but lacking an ER-targeting signal peptide and thus synthesized in the cytosol. ATG genes now appear to play a role in the underlying pathways, leading to the emergence of the concept of secretory autophagy [56]. To date, at least 41 ATG genes have been identified. Among these proteins, ATG-5 plays a critical role in autophagic vesicle formation [57]. However, further research is needed to determine whether ATG-5 alone can serve as a reliable and sufficient biomarker of autophagic activity.

Circadian timing plays a key role in the metabolic benefits of daytime-restricted intermittent fasting. Organisms have evolved internal clocks that align physiological processes with day-night cycles. TRF regimens reinforce diurnal eating patterns, which may enhance circadian gene expression and improve energy metabolism and weight regulation. Cortisol and melatonin are key hormonal markers of circadian rhythm [58]. Elevated melatonin during food intake may impair insulin secretion and glucose tolerance, suggesting that avoiding eating 2–3 h before sleep could offer metabolic benefits [59]. Daytime-restricted feeding has been linked to lower morning cortisol levels. Early TRF, such as skipping dinner, may improve sleep through reduced nighttime cortisol and enhance alertness with higher morning levels [60]. One study found that early evening fasting reduced obesity risk, while late evening fasting increased it [61]. These results highlight the need for further research comparing early vs. late eating windows in socially acceptable contexts.

Fasting blood glucose, lipid profile, and liver enzyme levels before and after the intervention were analyzed as secondary outcomes of our study. Elevated aminotransferase tests are among the most common reasons for patients to consult gastroenterology or hepatology clinics. A meta-analysis (n = 35 studies) compiling the recent studies on blood lipids showed that intermittent fasting and energy-restricted diet interventions led to significant decreases in total cholesterol, LDL-C, and triglyceride concentrations. In contrast, HDL-C concentrations were not affected [62]. In our study, in parallel with the literature, fasting blood glucose, triglyceride, LDL-C, total cholesterol, ALT, and GGT levels decreased significantly in both energy-restricted and energy + time-restricted diet groups compared to the pre-intervention period. However, post-intervention total cholesterol changes in the energy + time-restricted diet group were significantly higher than in the energy-restricted diet group. In total, 3 of the 18 studies included in another meta-analysis evaluating the efficacy of intermittent fasting compared to continuous energy restriction in overweight and obese individuals found that intermittent fasting strategies were more effective in improving lipid profile. Some authors suggest that the mechanisms responsible for the effects of intermittent fasting on lipidaemia are related to metabolic adaptation to fasting. During fasting, glucose concentration decreases, and hepatic glycogen reserves are depleted, which activates gluconeogenesis and fatty acid oxidation. When glucose stores are depleted, the body utilizes ketones to convert fatty acids released by adipocytes in lipolysis. These metabolic processes can lead to an improvement in plasma lipid concentration as well as weight loss [63].

While weight loss remains the most effective treatment for MAFLD, the relationship between dietary components and MAFLD has recently drawn increasing interest [64]. A study investigating nutritional status in individuals with MAFLD (n = 106) found that saturated fatty acid intake was associated with advanced fibrosis levels [65]. Our study supports these findings; at T8, the proportion of energy from fat and intake of saturated fat decreased in both the energy + time-restricted diet group and the energy-restricted group. In addition, increased dietary fiber is beneficial in managing MAFLD by regulating intestinal permeability and protective effects against insulin resistance [65,66]. This finding is also supported by the results of our study demonstrating increased fiber intake in both the energy + time-restricted diet group and the energy-restricted group at T8. Our results also showed a significant increase in fructose intake in both groups. This may be attributed to the increased intake of fructose from fruits, which contain beneficial phytochemicals, micronutrients, and fibers that provide long-term satiety and healthy gut microbiota, and the fact that none of the patients consumed > 50 g of fructose per day, which has been reported to have no detrimental effect on lipid and glucose control [67,68].

Our study has several limitations. First, it did not use biopsy to detect hepatic steatosis. Although liver biopsy is considered the gold standard for diagnosis, it was not used in our study because it is invasive and involves high costs and complications. As mentioned above, circadian timing may play a significant role in determining the effects of overnight fasting on fatty liver; however, unfortunately, circadian rhythm biomarkers such as melatonin and cortisol were not measured. Additionally, a limitation of the study is the assessment of autophagy based solely on the levels of ATG-5 and Beclin-1, without evaluating other key markers such as p62/SQSTM1, which may affect the comprehensiveness and generalizability of the findings. In our study, factors such as the fact that our patients were within a specific BMI range and the study period was limited to 8 weeks may restrict the generalization of our results to individuals with more severe stages of MAFLD or advanced fibrosis groups. There are also potential confounding factors that may affect the interpretation of our study results. Other factors that may affect metabolism, such as participants’ physical activity levels, sleep patterns, and stress levels, were not assessed in detail. Since these variables may indirectly affect the metabolic effects of intermittent fasting, they should be addressed in more detail in future studies. It should also be noted that individual participants’ metabolic responses may vary and that genetic factors may also affect the response to nutritional interventions.

## 5. Conclusions

The present study found that intermittent fasting may positively affect hepatic steatosis as determined by transient elastography. Our results confirm that patients with MAFLD can be prescribed intermittent fasting (time-restricted eating) diets under the guidance of a specialized dietitian, considering patients’ medical history. Future studies with biopsy evidence, larger samples, and longer duration may help better evaluate this effect’s clinical significance.

In daily clinical practice, the intermittent fasting model can also be considered as an option when planning dietary intervention in overweight or obese MAFLD patients. In particular, the 16:8 time-restricted feeding pattern can potentially improve liver health by promoting weight loss. In addition, the ability of the patient to customize the feeding window (for example, between 12:00 and 20:00 or 10:00 and 18:00) may be a factor that increases dietary compliance by providing flexibility. However, a personalized nutrition plan should be developed for each individual, considering their metabolic status, lifestyle, and adaptive capacity.

## Figures and Tables

**Table 1:** Analysis of anthropometric and routine biochemical measurements after intervention.

		Energy + Time-Restricted Diet Group	Energy-Restricted Diet Group	*p* (Inter-Groups) ^β^
Anthropometric Measurements				
Body weight (kg)	T0	88.9 (78.0–97.0)	93.0 (82.4–103.0)	**0.01**
	T8	83.0 (74.6–89.5)	88.0 (79.9–100.1)	
	*p* (Intra-groups) ^α^	**<0.001**	**<0.001**	
BMI (kg/m^2^)	T0	32.8 (29.2–35.2)	33.6 (30.2–37.3)	**0.01**
	T8	30.6 (27.3–33.8)	32.4 (29.1–35.7)	
	*p* (Intra-groups) ^α^	**<0.001**	**<0.001**	
Waist circumference (cm)	T0	105.0 (96.7–110.2)	107.0 (100.7–116.5)	**0.03**
	T8	99.0 (92.0–103.2)	101.0 (96.5–113.0)	
	*p* (Intra-groups) ^α^	**<0.001**	**<0.001**	
Waist–hip ratio	T0	0.95 (0.87–0.97)	0.95 (0.88–0.99)	0.09
	T8	0.92 (0.85–0.94)	0.93 (0.88–0.98)	
	*p* (Intra-groups) ^α^	**0.02**	0.06	
Body fat mass (kg)	T0	31.5 (25.5–33.9)	29.8 (25.5–40.0)	**0.01**
	T8	25.5 (21.6–30.4)	27.4 (23.6–36.9)	
	*p* (Intra-groups) ^α^	**<0.001**	**<0.001**	
Body fat percentage (%)	T0	34.5 (28.7–38.8)	34.2 (28.8–38.8)	**0.04**
	T8	31.9 (25.2–37.2)	32.9 (27.7–537.2)	
	*p* (Intra-groups) ^α^	**<0.001**	**<0.001**	
Fat-free mass (kg)	T0	52.8 (48.9–63.9)	57.9 (50.1–65.9)	0.22
	T8	51.7 (47.6–61.0)	55.1 (48.8–64.8)	
	*p* (Intra-groups) ^α^	**<0.001**	**<0.001**	
Biochemical Measurements				
Fasting Blood Glucose (mg/dL)	T0	105.0 (100.7–116.5)	106.0 (64.7–115.0)	0.68
	T8	98.5 (96.0–106.5)	100.0 (91.0–102.7)	
	*p* (Intra-groups) ^α^	**<0.001**	**<0.001**	
Triglyceride (mg/dL)	T0	167.0 (110.5–229.7)	183.0 (122.7–200.0)	0.48
	T8	147.5 (101.0–201.7)	167.0 (106.0–188.0)	
	*p* (Intra-groups) ^α^	**<0.001**	**<0.001**	
LDL-C (mg/dL)	T0	154.0 (126.6–180.5)	150.0 (130.3–171.0)	0.86
	T8	135.0 (108.7–145.0)	127.0 (107.2–150.2)	
	*p* (Intra-groups) ^α^	**<0.001**	**<0.001**	
HDL-C (mg/dL)	T0	43.0 (33.8–48.5)	38.0 (32.0–47.9)	0.32
	T8	43.0 (33.7–46.7)	38.5 (33.7–46.7)	
	*p* (Intra-groups) ^α^	0.07	0.17	
Total Cholesterol (mg/dL)	T0	213.0 (175.7–242.7)	219.0 (200.7–251.7)	**0.01**
	T8	187.0 (144.5–209.7)	199.50 (168.7–209.5)	
	*p* (Intra-groups) ^α^	**<0.001**	**<0.001**	
AST (IU/L)	T0	35.9 (20.8–68.0)	25.7 (21.7–39.2)	**0.01**
	T8	31.5 (21.0–47.5)	25.5 (20.7–35.5)	
	*p* (Intra-groups) ^α^	**<0.001**	0.09	
ALT (IU/L)	T0	38.7 (34.1–69.7)	39.0 (36.7–67.0)	0.29
	T8	35.0 (24.5–53.5)	35.0 (31.0–48.7)	
	*p* (Intra-groups) ^α^	**<0.001**	**<0.001**	
GGT (IU/L)	T0	46.7 (31.0–70.0)	45.0 (31.5–56.5)	0.22
	T8	38.5 (26.7–52.7)	34.5 (26.2–46.5)	
	*p* (Intra-groups) ^α^	**<0.001**	**<0.001**	

Continuous variables are expressed median (Quartile 1–Quartile 3). *p* (Intra-groups) ^α^: Wilcoxon analysis was performed; *p* (Inter-groups) ^β^: Mann–Whitney U test was performed. Statistical significance, *p* < 0.05, significant *p* values are indicated in bold. ALT: alanine aminotransferase, AST: aspartate aminotransferase, BMI: body mass index, GGT: gamma-glutamyl transferase, HDL-C: high-density lipoprotein cholesterol; LDL-C: low-density lipoprotein cholesterol.

**Table 2:** Correlation between FGF-21 and autophagy markers.

		FGF-21 (pg/mL)	ATG-5 (ng/mL)	Beclin-1 (ng/mL)
FGF-21 (pg/mL)	R		0.343	−0.030
	*p*		**0.02**	0.84
ATG-5 (ng/mL)	R	0.343		0.342
	*p*	**0.02**		**0.02**
Beclin-1 (ng/mL)	R	−0.030	0.342	
	*p*	0.84	**0.02**	

Spearman’s correlation was performed. Significant *p* values are indicated in bold. ATG-5; autophagy-related protein-5; FGF-21; fibroblast growth factor-21.

**Table 3:** FGF-21 and autophagy marker correlation with liver disease parameters.

		FGF-21 Change (pg/mL)	ATG-5 Change (ng/mL)	Beclin-1 Change (ng/mL)	CAP Change (dB/m)	LSM Change (kPa)	AST Change (IU/L)	Body Weight Loss (%)
FGF-21 change (pg/mL)	R		0.089	−0.013	0.043	−0.029	0.209	0.167
	*p*		0.54	0.93	0.77	0.84	0.15	0.25
ATG-5 change (ng/mL)	R	0.089		−0.218	0.131	0.069	0.029	0.095
	*p*	0.54		0.14	0.37	0.64	0.84	0.52
Beclin-1 change (ng/mL)	R	−0.013	−0.218		−0.029	−0.164	0.056	−0.056
	*p*	0.93	0.14		0.84	0.27	0.71	0.71
CAP change (dB/m)	R	0.043	0.131	−0.029		0.277	0.085	0.355
	*p*	0.77	0.37	0.84		0.05	0.56	**0.01**
LSM change (kPa)	R	−0.029	0.069	−0.164	0.277		0.094	0.364
	*p*	0.84	0.64	0.27	0.05		0.52	**0.01**
AST change (IU/L)	R	0.209	0.029	0.056	0.085	0.094		0.211
	*p*	0.15	0.84	0.71	0.56	0.52		0.15
Body Weight Loss (%)	R	0.167	0.095	−0.056	0.355	0.364	0.211	
	*p*	0.25	0.52	0.71	**0.01**	**0.01**	0.15	

Spearman’s correlation was performed. Significant *p* values are indicated in bold. CAP: controlled attenuation parameter; LSM: liver stiffness measurement; ATG-5: autophagy-related protein-5: FGF-21: fibroblast growth factor-21; AST: aspartate aminotransferase.

**Table 4:** Analysis of energy and nutrient intake after intervention.

		Energy + Time-Restricted Diet Group	Energy-Restricted Diet Group	*p* (Inter-Groups) ^β^
Energy (kcal)	T0	1882.7 (1596.2–2014.8)	1919.7 (1749.0–2193.0)	0.80
	T8	1577.0 (1488.0–1726.4)	1687.2 (1499.8–1796.5)	
	*p* (Intra-groups) ^α^	**<0.001**	**0.01**	
Carbohydrate (%)	T0	43.0 (37.5–48.0)	41.5 (38.0–50.2)	0.89
	T8	53.0 (50.7–55.0)	52.0 (50.7–53.0)	
	*p* (Intra-groups) ^α^	**<0.001**	**<0.001**	
Protein (%)	T0	15.0 (14.0–17.2)	16.0 (14.0–17.0)	0.63
	T8	19.0 (18.0–20.0)	19.0 (18.0–20.0)	
	*p* (Intra-groups) ^α^	**<0.001**	**<0.001**	
Fat (%)	T0	42.0 (36.7–46.0)	42.5 (37.5–46.2)	0.99
	T8	28.0 (26.0–30.0)	29.0 (27.0–30.0)	
	*p* (Intra-groups) ^α^	**<0.001**	**<0.001**	
Saturated fat (g)	T0	31.8 (25.9–37.9)	31.8 (29.7–36.4)	0.77
	T8	18.6 (16.3–19.1)	19.0 (16.7–20.3)	
	*p* (Intra-groups) ^α^	**<0.001**	**<0.001**	
Fiber (g)	T0	21.4 (15.8–25.2)	21.5 (18.2–26.2)	0.21
	T8	30.1 (27.2–31.2)	30.1 (28.6–31.2)	
	*p* (Intra-groups) ^α^	**<0.001**	**<0.001**	
Fructose (g)	T0	11.4 (6.3–20.3)	13.5 (5.0–20.8)	0.69
	T8	26.5 (21.0–27.6)	25.1 (24.6–26.9)	
	*p* (Intra-groups) ^α^	**<0.001**	**0.01**	

Continuous variables are expressed as median (Quartile 1–Quartile 3). *p* (Intra-groups) ^α^: Wilcoxon analysis was performed; *p* (Inter-groups) ^β^: Mann–Whitney U test was performed. Statistical significance, *p* < 0.05, significant *p* values are indicated in bold.

## Data Availability

The data obtained in this study are available from the corresponding author upon request. The data are not publicly available due to subjects’ privacy.

## Abbreviations

The following abbreviations are used in this manuscript:

ALT	Alanine Aminotransferase
AST	Aspartate Aminotransferase
ATG-5	Autophagy-Related Protein-5
BMI	Body Mass Index
CAP	Controlled Attenuation Parameter
FGF-21	Fibroblast Growth Factor-21
GGT	Gamma-Glutamyl Transferase
HDL-C	High-Density Lipoprotein Cholesterol
LDL-C	Low-Density Lipoprotein Cholesterol
LSM	Liver Stiffness Measurement
MAFLD	Metabolic Dysfunction-Associated Fatty Liver Disease
NAFLD	Non-Alcoholic Fatty Liver Disease
